# Metabolic profiling of the three neural derived embryonal pediatric tumors retinoblastoma, neuroblastoma and medulloblastoma, identifies distinct metabolic profiles

**DOI:** 10.18632/oncotarget.24168

**Published:** 2018-01-11

**Authors:** Sarah E. Kohe, Christopher D. Bennett, Simrandip K. Gill, Martin Wilson, Carmel McConville, Andrew C. Peet

**Affiliations:** ^1^ Institute of Cancer and Genomic Sciences, University of Birmingham, Birmingham, United Kingdom; ^2^ Birmingham Children’s Hospital, NHS Foundation Trust, Birmingham, United Kingdom; ^3^ Centre for Human Brain Health, School of Psychology, University of Birmingham, Birmingham, United Kingdom

**Keywords:** retinoblastoma, neuroblastoma, medulloblastoma, high resolution magnetic resonance spectroscopy, tumor metabolites

## Abstract

The rare pediatric embryonal tumors retinoblastoma, medulloblastoma and neuroblastoma derive from neuroectodermal tissue and share similar histopathological features despite different anatomical locations and diverse clinical outcomes. As metabolism can reflect genetic and histological features, we investigated whether the metabolism of embryonal tumors reflects their similar histology, shared developmental and neural origins, or tumor location. We undertook metabolic profiling on 50 retinoblastoma, 39 medulloblastoma and 70 neuroblastoma using high resolution magic angle spinning magnetic resonance spectroscopy (1H-MRS). Mean metabolite concentrations identified several metabolites that were significantly different between the tumor groups including taurine, hypotaurine, glutamate, glutamine, GABA, phosphocholine, N-acetylaspartate, creatine, glycine and myoinositol, *p* < 0.0017. Unsupervised multivariate analysis found that each tumor group clustered separately, with a unique metabolic profile, influenced by their underlying clinical diversity. Taurine was notably high in all tumors consistent with prior evidence from embryonal tumors. Retinoblastoma and medulloblastoma were more metabolically similar, sharing features associated with the central nervous system (CNS). Neuroblastoma had features consistent with neural tissue, but also contained significantly higher myoinositol and altered glutamate-glutamine ratio, suggestive of differences in the underlying metabolism of embryonal tumors located outside of the CNS. Despite the histological similarities and shared neural metabolic features, we show that individual neuroectodermal derived embryonal tumors can be distinguished by tissue metabolic profile. Pathway analysis suggests the alanine-aspartate-glutamate and taurine-hypotaurine metabolic pathways may be the most pertinent pathways to investigate for novel therapeutic strategies. This work strengthens our understanding of the biology and metabolic pathways underlying neuroectodermal derived embryonal tumors of childhood.

## INTRODUCTION

Pediatric solid tumors represent a diverse range of childhood cancers with unique anatomical location, cellular origins, and clinical presentation [1]. Tumors with an embryonic origin reflect a particular group of solid tumors that are derived from immature blast cells that fail to differentiate into normal tissue [1, 2]. Although these tumors can originate in many parts of the body, the most common locations in childhood include the liver (hepatoblastoma), kidney (nephroblastoma), sympathetic nervous system (neuroblastoma), brain (medulloblastoma), and eye (retinoblastoma). The current study investigates the metabolic profile of the three main childhood embryonal tumors that arise from the neuroectodermal tissue of the nervous system, namely retinoblastoma, medulloblastoma, and neuroblastoma.

Retinoblastoma is a tumor of the eye that develops from the precursor cells that form the retina. It occurs in very young children and has a good outcome with timely clinical intervention [3]. Neuroblastoma arises from the precursor cells of the neural crest-derived sympathetic nervous system, most commonly in the adrenal gland (60% of all cases) but can also develop from the sympathetic ganglia of the abdominal, thoracic, and pelvic regions [4, 5]. Although usually detected in children under two, neuroblastoma can also develop in older children, and has a heterogeneous clinical outcome [5]. Medulloblastoma is the most common malignant brain tumor of childhood and develops from the progenitor cells that form the cerebellum of the brain. Survival rates range from less than 45% to over 95% depending on molecular genetic subgroup [6]. Despite being very distinct clinical entities (Table 1), the microscopic appearance of these three tumor groups is often indistinguishable and they characteristically show a resemblance to tissue of the immature developing nervous system [7].

**Table 1:** Population demographics of each tumor type and the characteristics of the samples included in this study

	Retinoblastoma	Medulloblastoma	Neuroblastoma
Population and clinical characteristics			
**Anatomical location**	eye	brain (cerebellum)	sympathetic nervous system (various locations)
**Mean age at diagnosis**	18 months	5–6 years	2–3 years
**% of all childhood cancers** **(0–14y) ; USA**^a^**UK** ^b^	3% (280 per year) 3% (44 new cases per year)	3.8% (400 per year) 3.5% (54 new cases per year)	7% (710 per year) 6% (78 new cases per year)
**Gender distribution (F:M);** **Range: USA**^a^**-UK** ^b^	48–50%	60–65%	53–55%
**5Y overall survival rate;** **Range: USA**^a^**-UK** ^b^	99%	65–71.3%	65%–79%
**Characteristics of tumor samples included in this study**			
**Sample numbers**	*n* ***=*** 50	*n* ***=*** 39	*n* ***=*** 70
**Period samples collected over**	2004–2013	1999–2013	1996–2012
**Mean age at diagnosis (years)**	1.5	6.5	2.5
**Gender distribution**	37% male	75% male	60% male
**5Y survival rate**	100%	60%	67%

^a^Reported in Mathew et al., Neuro-oncology 2014 [59] ^b^Statistics reported on CRUK website ^c^Reported in Ward et al., CA Cancer J Clin 2014 [60].

The presence of characteristic histopathological features such as rosettes is often evident, including Homer-Wright rosettes which represent neural differentiation and Flexner-Wintersteiner rosettes which are a feature of retinoblastoma [7, 8]. The ectoderm, or external germ layer of the embryo, forms the basis of the nervous system including the spine, retina, brain, and peripheral nervous system, along with skin [9]. Although all three tumors develop from primitive neuroectodermal precursor tissue [1], structures of the central nervous system (CNS) including the cerebellum and retina arise from the neural tube, whilst the peripheral nervous system develops from the adjacent neural crest [9]. The tissue metabolic profile is known to reflect the molecular genetic and histological features of individual tumors [10], however it is unknown to what extent neural origin, tumor location, and developmental processes influence the metabolism of embryonal childhood tumors.

Ex vivo metabolite profiling of tumor tissue with high resolution magic angle spinning magnetic resonance spectroscopy (1H-MRS) has identified diagnostic and prognostic metabolite markers in a range of tumors [11]. We have previously shown medulloblastoma can be discriminated from other childhood brain tumors using 1H-MRS, as well as evaluating glutamate as a prognostic marker [12, 13]. Furthermore, we have identified three main metabolic subgroups within retinoblastoma that correlated with histopathology and clinical features [14]. Metabolic profiling can also discriminate between MYCN-amplified and non-amplified neuroblastoma cell lines [15] and previous work by others has shown that it may be a useful method to distinguish stage [16]. These prior studies suggest that embryonal-derived tumor groups may share characteristic metabolic features associated with neural and neuroectodermal origin.

Tumors and brain disorders that occur within the CNS often contain imbalances in metabolites related to brain function. Prior research has linked the expression of N-acetyl Aspartate (NAA) and aspartate to both normal brain tissue and brain tumors [17]. NAA is known to be a marker of neuron number and integrity in normal brain, and is not found at high concentrations outside of the nervous system. Myoinositol is also characteristically seen in brain tumor tissue, particularly in tumors of glial origin [12, 18]. Interestingly myoinositol, creatine and NAA have been reported as the most important metabolites for discriminating brain region, further emphasizing their importance as neural metabolic markers [19]. Also of interest is the relationship between GABA, glutamate, and glutamine, which are classically regarded as neurotransmitters, but are known to independently influence tumor metabolism via the glutamate-glutamine cycle in cancer. Prior evidence indicates that taurine is notably elevated in primitive embryonal tumors, particularly those that are more differentiated [12, 14]. Although it is important in the developing brain, the function of taurine is largely unknown, aside from a role in neuroprotection.

The influence of tumor location on metabolic profile may also be important. Medulloblastoma forms within the cerebellum, which is rich in metabolites related to neural function, and is likely to reflect this neural origin. Normal retina is one of the most metabolically active tissues in the body, with high levels of metabolites related to increased energy demands. Neuroblastoma most commonly arises within the adrenal medulla, which has high levels of glutamate, myoinositol, scylloinositol, catecholamines and acetate [20]. Although a proportion of neuroblastomas arise in the sympathetic ganglia at other locations, evidence suggests that sympathetic ganglia and adrenal medulla develop along a shared sympathoadrenal pathway and retain similar markers [21].

Deregulation of choline metabolism is considered to be a hallmark of most cancers and treatments targeting these pathways have shown promise in slowing cell proliferation and reducing tumor growth [22]. Choline metabolites and in particular phosphocholine (PC), are considered markers of malignancy, with elevated levels reported in more aggressive tumors and in comparison to normal tissue [11, 23, 24]. The relationship between choline metabolites is also of interest, with differences in the ratio of glycerophosphocholine (GPC) to PC reflecting different underlying molecular alterations in breast tumors [25], and high GPC indicative of a less aggressive tumor in brain [11, 12, 22]. Glycine is associated with high grade in brain tumors [26], and is also elevated in many other tumors [24, 27]. Recent evidence has shown that rapidly proliferating cells display an increased reliance on glycine consumption and synthesis [28]. Although creatine is an important component of normal brain, prior studies have also associated increased creatine with increased energy metabolism in high grade brain tumors [11, 29]. Interestingly, creatine has been found to be particularly high in tumors of neuroectodermal origin [29].

Although many studies have investigated the tissue metabolic profile of medulloblastoma, retinoblastoma, and neuroblastoma individually, no study has yet compared the metabolomics of these three childhood embryonal tumors together. Previous studies have largely concentrated on identifying clinically relevant tumor subgroups and prognostic markers to benefit clinical decision-making. The purpose of this study was to investigate the comparative metabolic profiles of these tumors and elucidate if the metabolic profile reflects the neural features typical of neuroectodermal origin, the location that the tumor arises from, or developmental metabolic processes. We quantified and analyzed 1H-MRS tumor spectra acquired from 159 retinoblastoma, neuroblastoma and medulloblastoma. This presents a unique and rare opportunity to directly compare metabolism in three rare pediatric tumors using the same methods and will further our understanding of the metabolism of neural-derived embryonal tumors and the factors that influence metabolic profile in childhood tumors.

## RESULTS

**Figure 1:**
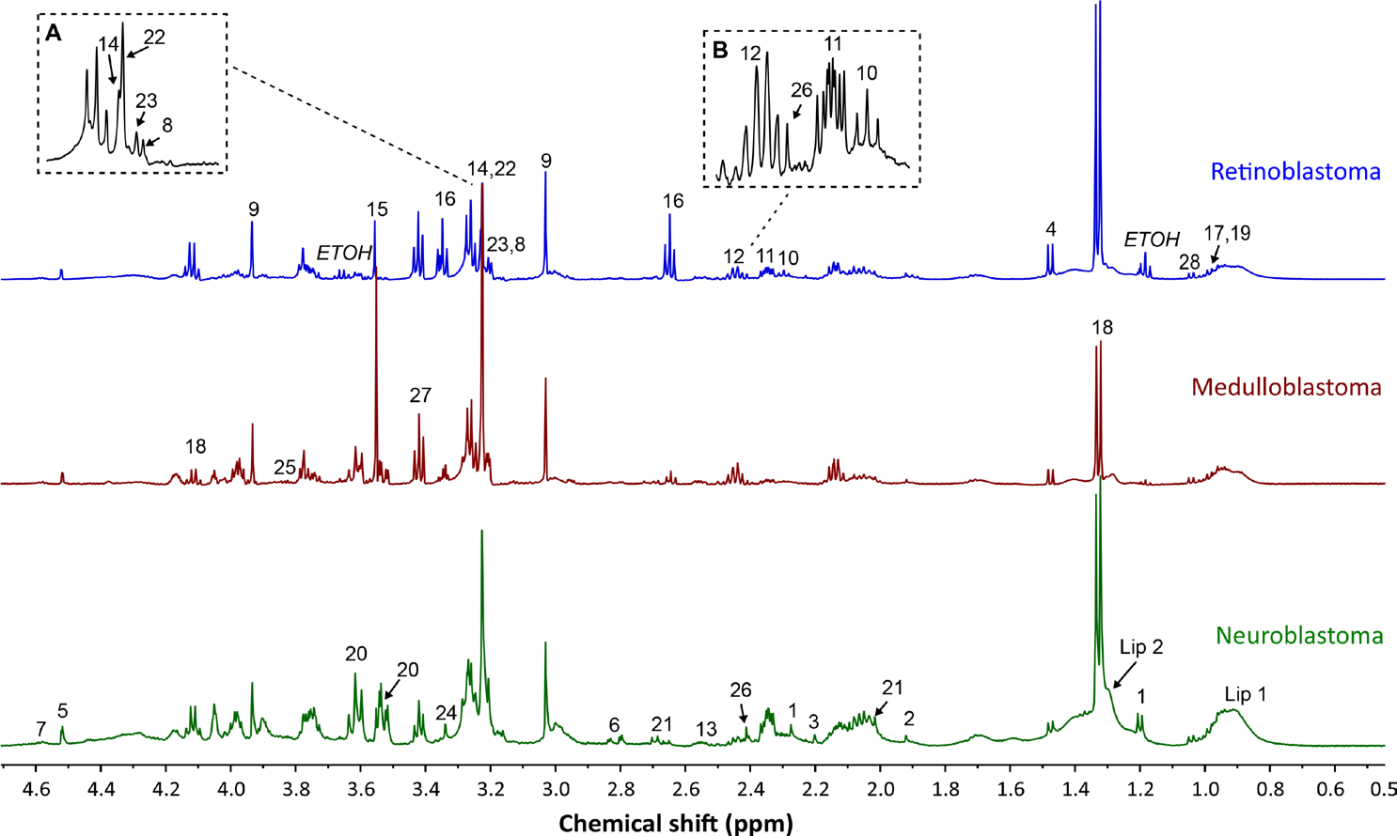
Example spectra from each tumor group, retinoblastoma, medulloblastoma, and neuroblastoma Assigned metabolites are labelled on relevant spectra; 3-hydroxybuturate (1), acetate (2), acetone (3), alanine (4), ascorbate (5), aspartate (6), beta-d-glucose (7), choline (8), creatine (9), GABA (10), glutamate (11), glutamine (12), glutathione (13), glycerophosphocholine (14), glycine (15), hypotaurine (16), isoleucine (17), lactate (18), leucine (19), myoinositol (20), NAA (21), phosphocholine (22), phosphoethanolamine (23), scylloinositol (24), serine (25), succinate (26), taurine (27), valine (28), lipid (Lip 1: 0.9ppm component and Lip 2: 1.3ppm component). (**A**) Detailed view of the choline region. (**B**) Detailed view of the glutamate/glutamine region. *Note; each metabolite has been labelled on only one of the example spectra, to improve the clarity of the figure.*

**Figure 2:**
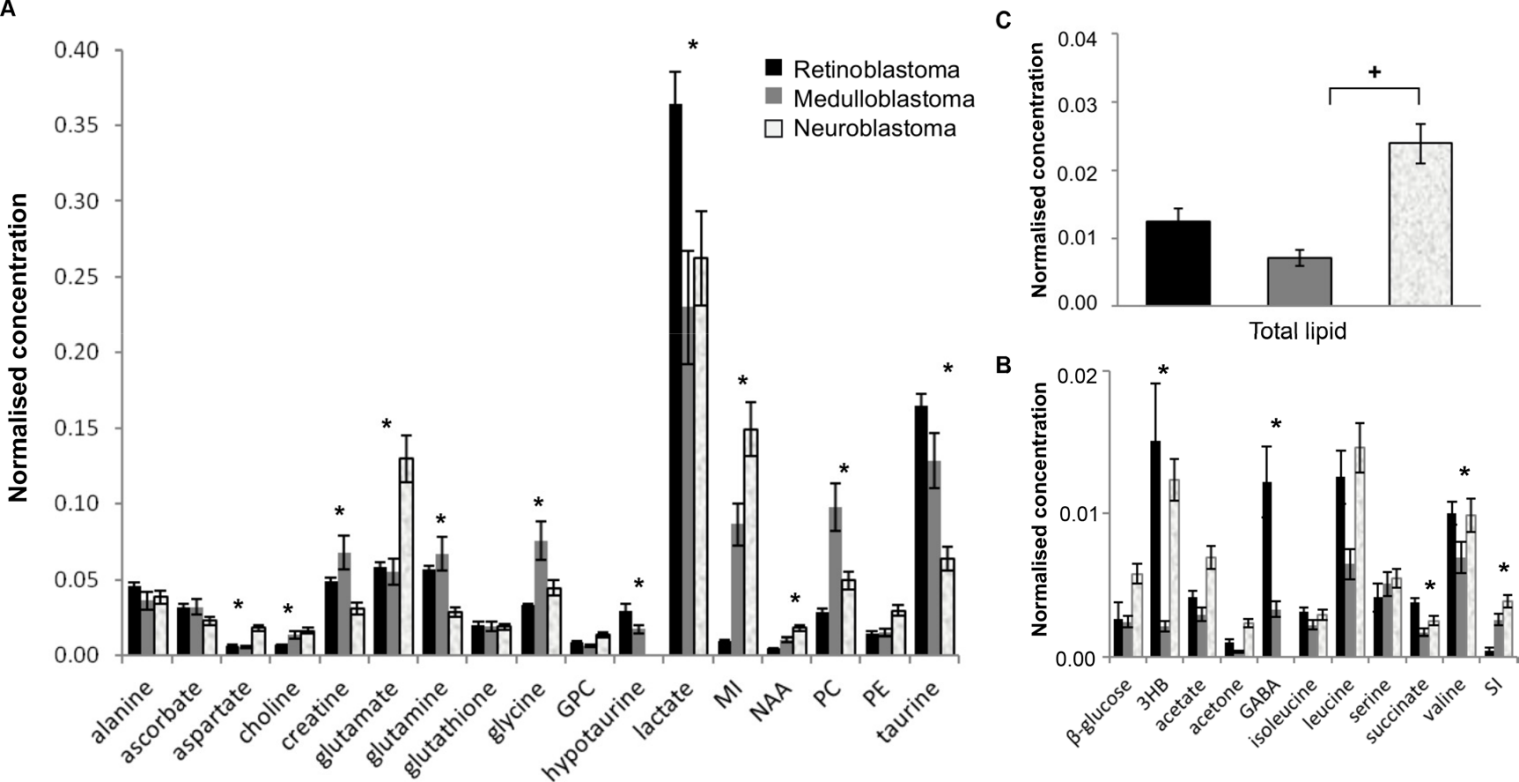
Differences in mean metabolite concentrations between retinoblastoma (*n* = 50), medulloblastoma *(n* = 39), and neuroblastoma (*n* = 70) (**A**) High concentration metabolites. (**B**) Low concentration metabolites. Data expressed as normalized mean concentration ± standard error, SEM. ^*^ indicates statistical significance detected between tumor groups with Kruskall-Wallis, *p* < 0.0017 (Bonferroni corrected). (**C**) Mean total lipid concentration, *p* = 0.001 Kruskall-Wallis, ^+^statistical significance between groups with Mann-Whitney *U* post hoc test, *p* < 0.05. PE; phosphoethanomine, MI; myoinositol, SI; scylloinositol.

Good quality spectra suitable for quantification purposes were available for 50 retinoblastoma, 39 medulloblastoma and 70 neuroblastoma. All samples had a metabolite profile consistent with that expected of tumor. Twenty-eight metabolites were routinely detected and assigned within tumor spectra (Figure 1). Analysis of mean metabolite concentrations identified 12 metabolites present at high concentrations (normalized mean concentration > 0.01) that were significantly different including aspartate, choline, phosphocholine (PC), creatine, glutamate, glutamine, glycine, hypotaurine, taurine, lactate, myoinositol, and NAA (Figure 2A, Supplementary Table 1). A further five low concentration metabolites (normalized mean concentration < 0.01) were also significantly different, including 3-hydroxybuturate (3HB), GABA, succinate, valine and scylloinositol (Figure 2B, Supplementary Table 1). Lactate was the highest concentration metabolite present in all tumor groups. Although it is often excluded when comparing small tumor groups as it is sensitive to post-biopsy metabolic degradation [10], our study suggests that despite increased variability in its concentration, there still appears to be a large difference in lactate concentration when considering these tumor groups as a whole. Lipids were observed in all three tumor groups, although mean concentration was particularly elevated in neuroblastoma (Figure 2C), interestingly high lipids in individual cases were generally associated with adverse clinical outcome.

### All three tumors display metabolic features characteristic of neural tissue

NAA and aspartate were detected in all three tumor groups, consistent with these metabolites being markers of neural-derived tissue [30]. The concentration of both NAA and aspartate in this study was highest in neuroblastoma, followed by medulloblastoma and retinoblastoma. NAA has been detected at low levels in neuroblastoma and normal adrenal medulla previously [16]. As expected, very low levels of NAA were seen in medulloblastoma compared to that evident in normal brain [23]. A markedly elevated mean concentration of myoinositol was evident in neuroblastoma, with moderate amounts in medulloblastoma and very little detected in retinoblastoma, *p* < 0.0001, Figure 2A, Supplementary Table 1. Although typically described as a glial marker it is unclear what the significance of myoinositol is in neuroblastoma. Creatine is often reported as a marker of neural tissue, and has been used to discriminate brain tumors with high energy demands. It was present in all three tumor groups, consistent with prior evidence of increased levels in neuroectodermal tumors [29, 31] but was significantly higher in medulloblastoma (*p* < 0.0017). Other neural markers such as GABA and glutamate were evident at varying concentrations in the three tumors. These metabolites play an important role in cancer metabolism in many tumor types throughout the body independent of their role in neural tissue [31, 32]. Therefore their concentration may be influenced by tumor location and the malignant properties of the tumor groups rather than solely as neural metabolites.

### Taurine is associated with both neural and developmental origins in embryonal tumors

Although taurine has been routinely identified in many types of brain tumors previously, the concentration of taurine was particularly prominent in all three tumor groups in this study. Elevated taurine has been previously detected in tumors of neuroectoderm embryonal origin relative to both its concentration in normal brain and in other non-embryonal brain tumors [12, 29]. A significant difference in concentration between each tumor was evident, with retinoblastoma having the highest concentration, followed by medulloblastoma, then neuroblastoma, *p* < 0.0001, Figure 2A, Supplementary Table 1. The concentration of hypotaurine, followed the pattern evident with taurine concentration, however it was absent in neuroblastoma, resulting in a hypotaurine to taurine ratio of 0, which was significantly decreased compared to retinoblastoma (0.173 ± 0.03, mean ± SEM) and medulloblastoma (0.210 ± 0.03), *p* < 0.0001.

### Tumor location influences metabolic profile

Both the location of the tumor itself and the function of the normal tissue that each tumor derives from, appears to influence metabolic profile. Although taurine is elevated in embryonal tumors, the concentration of taurine present in retinoblastoma and medulloblastoma also appears to be influenced by the high concentration of taurine in normal retina and cerebellum [19, 33, 34]. Succinate was also significantly elevated in retinoblastoma compared to medulloblastoma and neuroblastoma, *p* < 0.0001. This is notable given that succinate has been proposed as a metabolic driver of angiogenesis and neovascularization in retina and retinal disease [35]. It was also evident that lactate is notably higher in retinoblastoma than the other tumors, *p* < 0.0001. Lactate is the highest concentration metabolite in the retina and is known to be a viable energy source for metabolism in retinal tissue [36]. The significantly higher expression of GABA within retinoblastomas, *p* < 0.0001, (Figure 2B, Supplementary Table 1), is likely influenced by the high concentration of GABA detected in normal retina. Furthermore, GABA was noted at similar concentrations in a small number of medulloblastomas (*n* = 4), which may reflect molecular genetic subgroup [37]. GABA has not been reported in normal adrenal medulla [20], and was not detected in neuroblastoma in this study. Although scylloinositol, has been reported as a marker of poor prognosis in individual cases, the 3 fold increase in mean concentration in neuroblastoma and medulloblastoma compared to retinoblastoma (*p* < 0.0001), may be influenced by the metabolic profile of normal adrenal medulla and cerebellum. Scylloinositol has been detected in these locations previously [16, 20, 33] however it does not appear to be present in normal ocular tissue [34]. The catecholamine’s typically seen in adrenal medulla were undetectable in neuroblastoma, consistent with a prior study [16, 20].

### Differences in metabolites are characteristic of individual tumors and their proliferative capabilities

**Figure 3:**
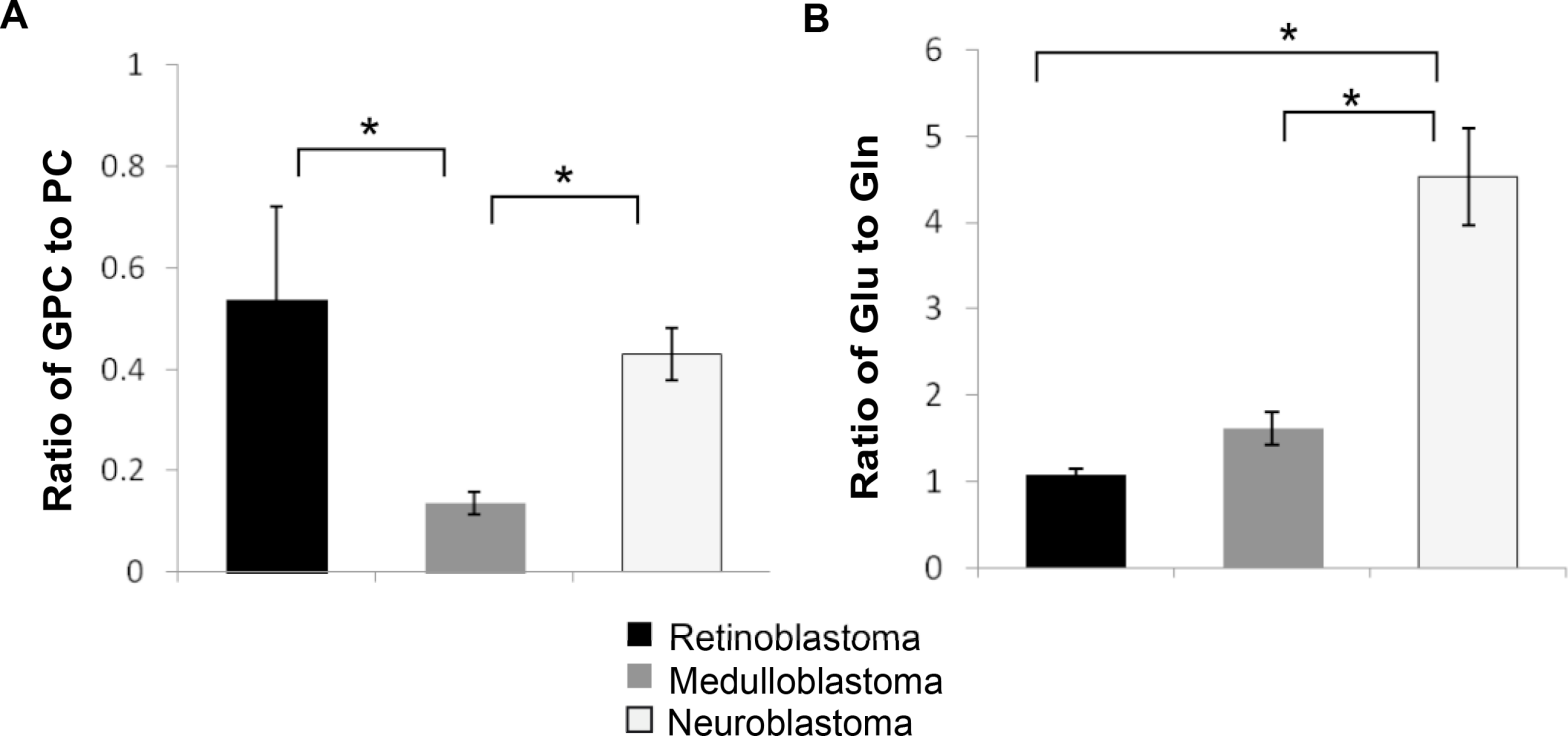
Metabolite concentration ratios differ between tumor groups (**A**) The ratio of GPC to PC and (**B**) the ratio of glutamate to glutamine is significantly different between tumor groups, *p* = 0.0001, Kruskall-Wallis, ^*^indicates statistical significance detected between groups with Mann-Whitney *U* post hoc test, *p* < 0.05. Data expressed as mean ratio ± SEM.

A number of metabolites were identified that are characteristic of tumors in general including elevated PC, glycine, and scylloinositol. Unsurprisingly PC was the second most highly concentrated metabolite in all three tumor groups after lactate, with the highest concentration in medulloblastoma followed by neuroblastoma, then retinoblastoma (*p* < 0.001, Figure 2A, Supplementary Table 1). GPC was unchanged between the three groups, however free choline was significantly elevated in both medulloblastoma and neuroblastoma compared to retinoblastoma, *p* < 0.0001. Differences in choline metabolism are also indicated by changes in the ratio of GPC to PC with a significantly lower ratio evident in medulloblastoma, 0.16, *p* < 0.0001, Figure 3A.

In addition to PC, medulloblastoma also had significantly increased mean concentration of glycine (*p* < 0.001) compared to the other two tumor groups. This suggests that both glycine and PC distinguish increased cellular proliferation and energy metabolism in medulloblastoma. Glutamate and glutamine are of particular interest in cancer metabolism. Interestingly neuroblastoma displayed the most significantly different glutamate-glutamine metabolism, with glutamate increased by 2-fold (*p* < 0.001) compared to the other tumors and glutamine reduced by half (*p* < 0.001). A markedly higher mean ratio of 4.71:1, of glutamate to glutamine was also evident, *p* < 0.0001, Figure 3B, (range: 2.23-11.21). In comparison the mean ratios for retinoblastoma and medulloblastoma were 1.07 and 1.18 respectively (Figure 3B).

The presence of lipid was variable in each tumor type, with neuroblastoma having a higher proportion of tumors with markedly elevated lipids, and a 2-fold higher mean lipid concentration than both retinoblastoma and medulloblastoma, *p* < 0.002 (Figure 2C). Interestingly, lipid was markedly elevated in all MYC and MYCN amplified neuroblastoma and medulloblastoma. The remaining medulloblastomas generally had very low concentrations. The mean concentration of lipid in MYC/MYCN tumors (5.65 ± 1.26, mean ± SEM) was significantly higher than in negative tumors (1.40 ± 0.22, *p* = 0.001).

### Metabolite differences can distinguish the different embryonal tumor groups

**Figure 4:**
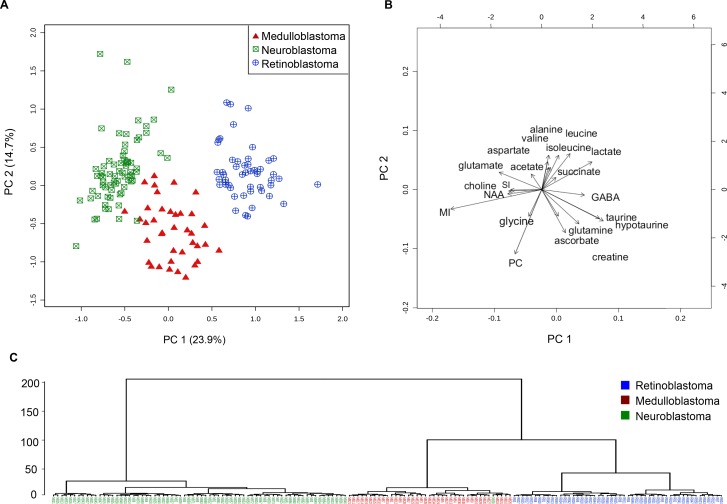
Embryonal tumor groups cluster separately in unsupervised multivariate analysis using metabolite concentrations (**A**) PCA scores plot demonstrating separate clusters for neuroblastoma (*n* = 70, squares), medulloblastoma (*n* = 39, triangles) and retinoblastoma (*n* = 50, circles) from left to right in principal component 1. (**B**) PCA scores biplot showing the metabolites elevated in each cluster. (**C**) Dendrogram analysis with Euclidian distance measure and Wards clustering method demonstrates that neuroblastoma tumors cluster to the left on their own branch. Medulloblastoma and retinoblastoma originate from the same right side branch, then divide and cluster separately. MI = myoinositol, SI = scylloinositol, PC = phosphocholine.

Next, we investigated the influence of tumor location, developmental and neural features, and individual tumor properties on tumor clustering in a multivariate analysis. Metabolite concentrations from each tumor were entered into a principal components analysis (PCA). Serine, acetone, phosphoethanomine and glutathione were excluded, as they were present at very low concentrations, and were not significantly different between tumor groups. The PCA showed little overlap between the three tumor groups (Figure 4A). Retinoblastoma and neuroblastoma were the most separated in principal component 1. Medulloblastoma appears to be more distinct in principal component 2, although this component is responsible for much less of the inter-tumor variation. Particular metabolites strongly influence the clustering of each tumor group within the PCA (Figure 4B). In retinoblastoma, lactate, succinate, GABA, and the branch chain amino acids valine, leucine and isoleucine are particularly elevated, whilst for medulloblastoma, glycine and PC are increased. Together, medulloblastoma and retinoblastoma have much higher taurine, hypotaurine, glutamine, creatine and ascorbate than neuroblastoma. Markedly elevated glutamate and myoinositol in comparison to the other groups were the most influential metabolites in neuroblastoma. Hierarchal dendrogram analysis identified almost complete separation between the three groups, with medulloblastoma and retinoblastoma clustering closely together and neuroblastoma appearing to be a more distinctly separate group.

### Relationships between metabolites have identified several key metabolic pathways that are altered

**Figure 5:**
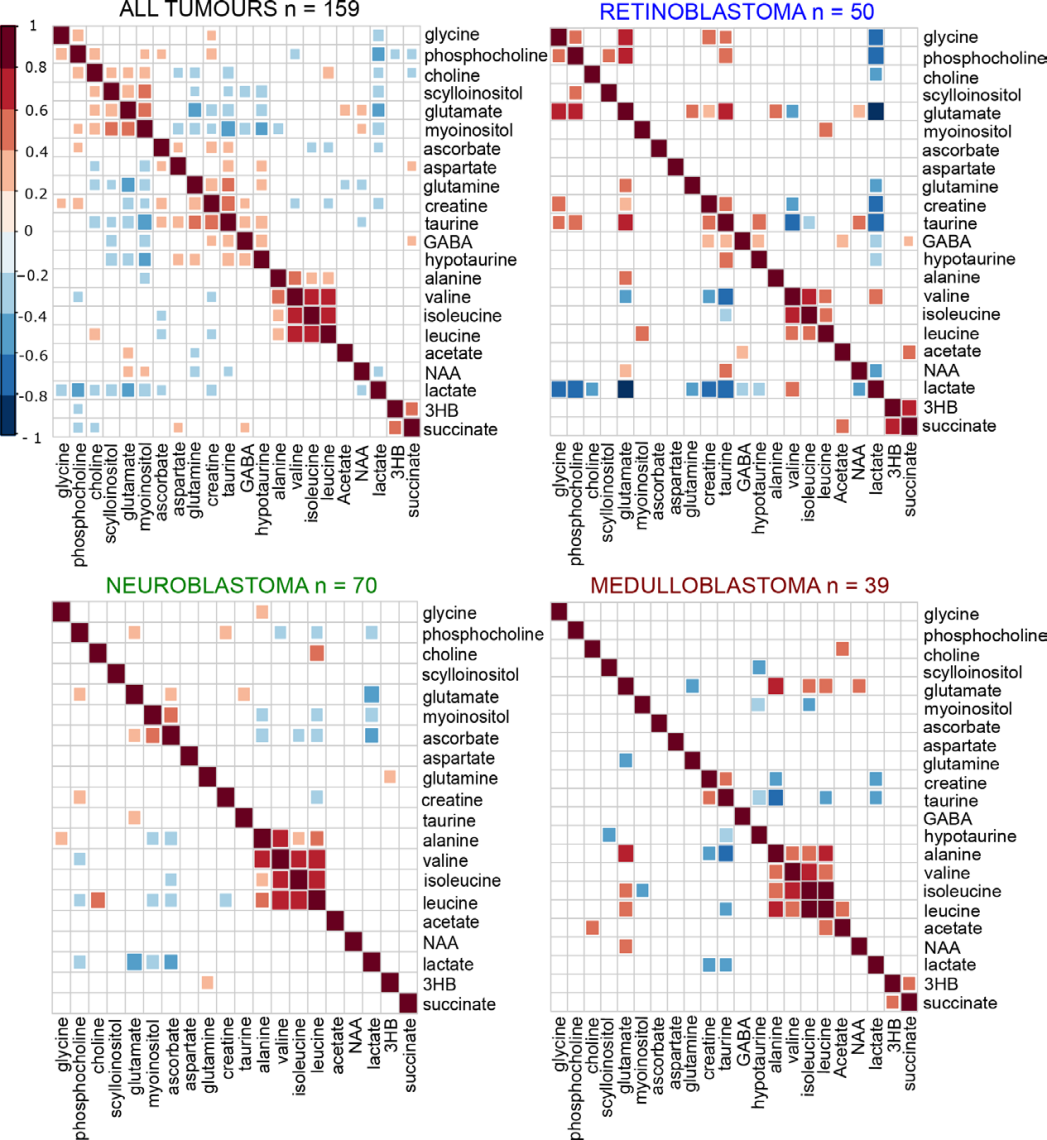
Differences in inter-metabolite correlations were evident between tumor groups Groups of highly correlated metabolites in the heat map containing all tumors (*n* = 159) are clustered together using hierarchal clustering. The individual tumor heat-maps were then ordered in a supervised manner according to the same pattern seen in the all tumors comparison, to identify if the similar patterns occurred in each tumor group. Dark red indicates a strong positive correlation between two metabolites (Pearson’s correlation *r* = 1 for a perfect positive correlation), dark blue indicates a strong negative correlation (Pearson’s correlation *r* = -1 for a perfect negative correlation). Only significant correlations are shown, non-significant correlations are left blank.

We also determined if differences were evident in metabolite correlations and metabolic pathways across the entire tumor cohort (Figure 5). As expected several pathway-related metabolites were positively correlated with each other including PC, GPC, and free choline, myoinositol and scylloinositol, taurine and hypotaurine, and the branched chain amino acids. Significant positive correlations were identified between metabolites previously associated with poor prognosis, including the choline metabolites, glutamate and scylloinositol. Glutamate and glutamine were negatively correlated with each other overall. When metabolite correlations were investigated within each individual tumor group, PC, glycine, and glutamate were strongly positively correlated in retinoblastoma and negatively correlated with taurine (Figure 5). In medulloblastoma, glutamate was positivity correlated with glycine, but negatively correlated with PC. Glutamine was strongly positively correlated with glutamate in retinoblastoma, negatively correlated in medulloblastoma, and not correlated in neuroblastoma. A similar pattern was evident with taurine and hypotaurine in retinoblastoma and medulloblastoma.

**Figure 6:**
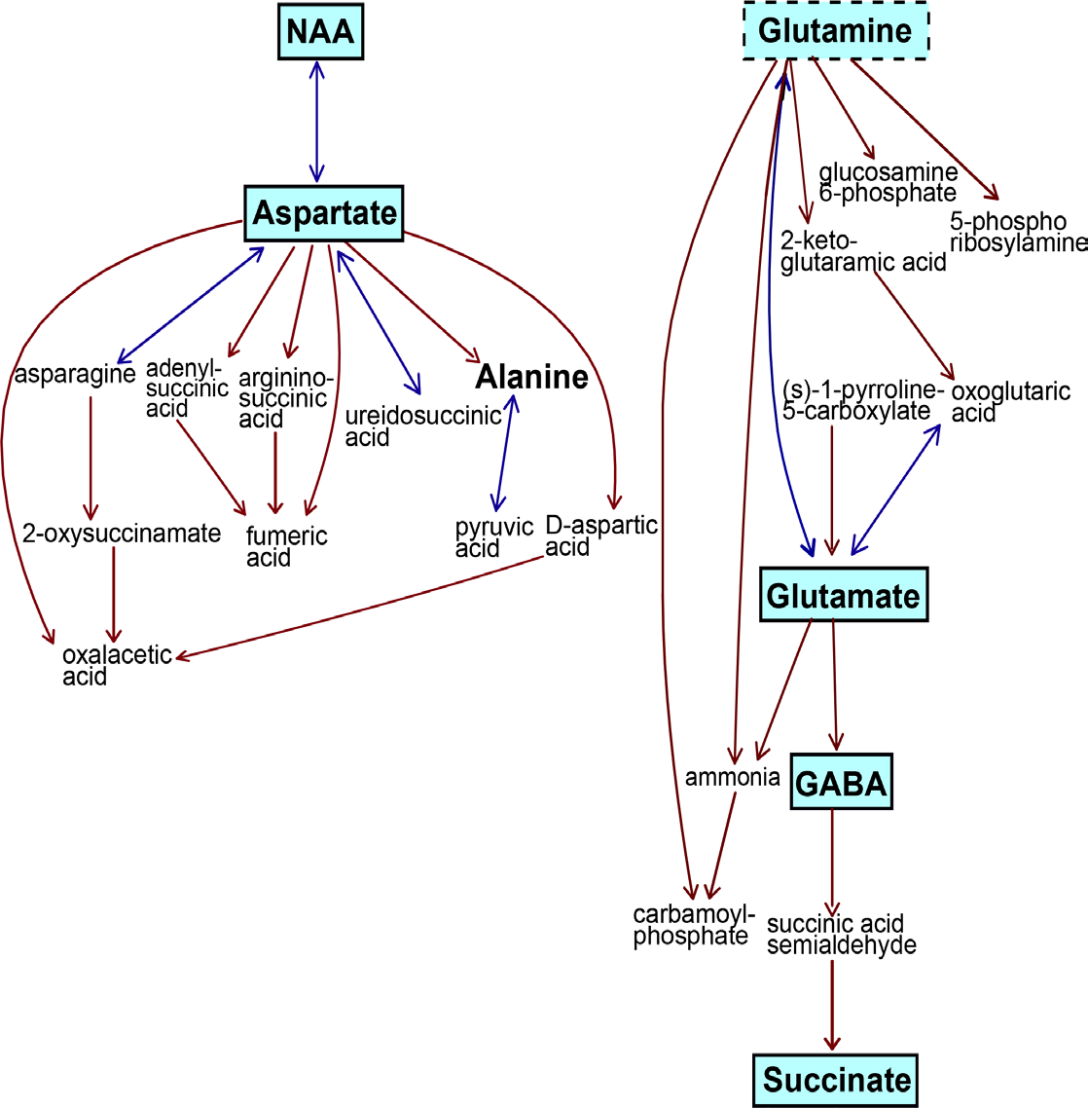
The alanine/glutamate/aspartate metabolic pathway was the most significantly different pathway between all three tumor groups Five metabolites were highly significant across this pathway, *p* < 0.0001(indicated by solid boxes with blue shading). Glutamine was also significant, *p* < 0.001, dotted box. Alanine was not significantly different. Metabolites in small text with no borders were not detected by 1H-MRS methods. This pathway is a modified metabolic pathway from KEGG database.

Pathway analysis showed the alanine, aspartate, and glutamate metabolic pathway was the most significantly altered between all three tumor groups with a pathway impact value of 0.75 out of 1, *p* = 0.00001 (Figure 6). The taurine and hypotaurine pathway was also significantly altered, with an impact value of 0.41, *p* = 0.0002. Other significantly different pathways included inositol phosphate metabolism (impact value 0.14, *p* = 0.00249), glycine, serine and threonine metabolism (0.19, *p* = 0.0001) and glycerophospholipid metabolism (0.19, *p* < 0.00239). Within each individual tumor group, the alanine, aspartate, glutamate metabolic pathway remained the most altered pathway, however the impact value was decreased in neuroblastoma (0.41) compared to retinoblastoma (0.70) and medulloblastoma (0.75).

## DISCUSSION

To our knowledge this is the first study to carry out a parallel investigation of these three neural derived embryonic tumors, retinoblastoma, medulloblastoma and neuroblastoma, using metabolomics. This study addresses the underlying influence of tumor location, origin and development on the metabolism of these three embryonal tumors. Metabolites related to neural origin were evident in all three tumors, with the greatest similarities noted between retinoblastoma and medulloblastoma. Nevertheless all three tumors clustered separately based on the complete metabolic profile (Table 2), emphasizing the unique features of each tumor type, related to their development and location.

**Table 2:** Summary of key metabolite differences between neural-derived embryonal tumors

	Retinoblastoma	Medulloblastoma	Neuroblastoma
**Taurine**	High	Intermediate	Low
**Hypotaurine**	High	Intermediate	Absent
**Glutamate**	Intermediate	Intermediate	High
**Glutamine**	Intermediate	Intermediate	Low
**GABA**	High	Low	Absent
**Phosphocholine**	Low	High	Intermediate
**Myoinositol**	Very low	Intermediate	High
**Glycine**	Low	High	Intermediate
**NAA**	Low	Intermediate	High
**Creatine**	High	High	Low

### Tumors derived from neuroectodermal tissue retain some neural features

Small amounts of NAA were evident in medulloblastoma, indicating that these tumors do retain neural metabolic features, perhaps unsurprising given that brain tumors often contain NAA. It is possible that the NAA reflects trapped neuronal elements within the tumor, although the concentration present within tumors was not consistent with the presence of large amounts of normal brain. Very little NAA was detected in retinoblastoma, possibly influenced by it being of much lower concentration in retina compared to other areas of the CNS [30]. Notably NAA, aspartate and acetate were all highest in neuroblastoma, suggesting that neuroblastoma shares features consistent with nervous tissue. However it is becoming evident that NAA may have a much wider role in tumors, with high levels associated with poor outcome in several tumors that occur outside of the nervous system [38]. Although NAA did not appear to be present in normal adrenal medulla, it has been detected in pheochromocytoma, a tumor that also derives from adrenal medulla. Creatine was elevated in medulloblastoma, perhaps reflecting not only increased energy metabolism in this tumor, but also that the cerebellum has been purported to contain the highest concentration of creatine when compared to other brain regions including frontal cortex, brainstem, and hippocampus [19].

Myoinositol is regarded as an osmolyte, with a possible role in cell signaling, although very little is known about its function. It is notably raised in the CNS, suggesting it has a particular role within neural tissue [18]. In pediatric brain tumors, notably elevated myoinositol is evident in ependymoma compared to pilocytic astrocytomas and medulloblastoma [39]. Elevated myoinositol has not been previously reported in neuroblastoma tissue, although an association with patients diagnosed at younger ages (< 2Y) was noted by Imperiale [16]. We found a moderate concentration of myoinositol in medulloblastoma consistent with previous studies [12, 39]. The almost complete absence in retinoblastoma suggests that it is not a feature of this tumor.

### Metabolic profile of each respective normal tissue influences tumor metabolic profile

The metabolic profile of medulloblastoma is influenced by its neural origins, and its location within the cerebellum of the brain. Cerebellum is known to contain high taurine, and creatine, and low myoinositol relative to other areas of the brain [19, 29, 33], consistent with the profile of medulloblastoma in the current study. Previous work investigating metabolism in normal retina obtained from post-mortem tissue has shown that it is particularly high in lactate, taurine, GABA, creatine, succinate, and the branched chain amino acids valine, leucine and isoleucine [34], likely reflecting its increased metabolic rate relative to other tissues within the body [40]. The metabolic profile of retinoblastoma was consistent with this. Although myoinositol has been detected at high concentration in normal adult retina [34], it was at very low concentration in retinoblastoma. It may be that myoinositol is present at higher concentrations in adulthood. Normal adrenal medulla has high levels of taurine, glutamate, myoinositol, scylloinositol, and the catecholamines adrenaline and noradrenaline, relative to adrenal cortex [20] which develops from the mesoderm rather than the neural crest [41]. The metabolic profile of neuroblastoma seems to closely reflect the profile of adrenal medulla. Furthermore, glutamate and myoinositol also appear to be present at high concentrations in pheochromocytoma and paragangliomas, tumors which also derive from the adrenal medulla [42].

### Metabolism in embryonal derived tumors is influenced by developmental processes

Taurine was elevated in all three tumor groups compared to non-embryonal tissue consistent with prior reports of it being present at higher concentrations in neuroectodermal derived tissue [12, 29]. A known cellular osmolyte, it is also a neurotransmitter, with taurine-specific synthesizing enzymes, receptors and transporters present within the CNS [43]. However it appears that taurine concentration is also influenced by developmental processes. It is known to be essential in neonates for normal brain development and depletion can result in long term deficits in brain function. A clear age gradient of brain taurine content is evident from mouse and human studies with concentration being much lower in adult brain than in early postnatal life [33, 44]. Higher concentrations of taurine may relate to the specific role of taurine within the early development of the retina and cerebellum. In retina it is particularly important for rod photoreceptor development from retinal precursor cells, whilst in the cerebellum it is important for correct migration of the precursor cerebellar granular neuronal cells [43]. It is also known to influence differentiation of neural precursor cells in these regions during development [45]. In many adult cancers, elevated taurine is associated with high grade and poor survival including pancreatic, colorectal, and prostate cancer [10]. However in retinoblastoma, it is more closely associated with the better differentiated clinicopathological sub-group, further emphasizing its role in differentiation during development [14]. Taurine was lower in neuroblastoma suggesting that it may not be as influenced by developmental processes occurring outside of the brain. Taurine content in individual neuroblastomas may indicate poor survival outcome, as shown by a previous study [16]. Although little is known about the role of hypotaurine, we have shown that the taurine/hypotaurine pathway is of importance and warrants further investigation, particularly in neuroblastoma.

### Differences in particular metabolites are associated with underlying clinicopathological risk factors

PC, a component of the total choline signal in tumors was also significantly different between all three tumor types. Changes in choline metabolism are typically associated with many types of malignancy. Medulloblastoma had the highest concentration of the three tumors, consistent with prior studies [12]. Although PC has not previously been associated with outcome in neuroblastoma patients, the lower mean concentration may reflect the wide range of stage and outcomes in the neuroblastoma cohort included in this study. Retinoblastoma had the lowest concentration of PC. Despite this, it is still known that PC correlates with adverse histology and greater clinical risk in retinoblastoma [14]. The ratio of GPC to PC may be an additional indicator of malignant status in brain tumors, with a higher ratio of GPC to PC linked to less aggressive behavior [25]. Tumor proliferation measured by Ki67 index is typically high in most medulloblastomas, but more variable across retinoblastoma and neuroblastoma. Whilst it is evident that altered choline metabolism likely reflects malignant behavior in each of the tumor groups, it seems apparent that high PC may also reflect the cellularity of the tumor, rather than solely an increase in relative aggressiveness between groups. Interestingly glycine, another marker of adverse outcome [26, 27], was also elevated in medulloblastoma. However unlike PC which was elevated in almost all medulloblastomas, glycine was notably raised in those cases with poor survival.

The role of glutamate and glutamine metabolism within these three tumor groups is also of interest, particularly in neuroblastoma where glutamine was at markedly low concentration, and glutamate was very high. A mean imbalance in glutamate is not evident in medulloblastomas as a whole, although it is apparent that high glutamate and altered ratios of glutamate to glutamine seems to be of prognostic significance [13]. Almost all neuroblastomas in this study had high glutamate, and in particular, an imbalance in the ratio of glutamate to glutamine, even those with low risk disease. There is strong evidence for altered glutamine metabolism being of diagnostic and prognostic significance in the neuroblastoma, and it is possible that there is change in flux within the glutamate-glutamine cycle [46]. This has been reported in other tumors and will require further investigation.

The identified association between high risk MYC and MYCN driven tumors and elevated lipids in medulloblastoma and neuroblastoma is also notable. Previous studies in lymphoma, lung and liver cancers have also reported changes in lipid composition, signaling and metabolism with MYC amplification [47–49], suggesting that the relationship between MYC and lipids is not restricted to tumors developed from neural derived tissue. Indeed in lymphoma, mass spectrometry imaging has identified a unique lipid signature that is characteristic of MYC driven disease, whilst non-MYC lymphoma has a different underlying lipid metabolic profile [47]. A similar finding has also been reported in lung tumors [48]. This may be important for specifically targeting inhibition of MYC driven tumors, which have a particularly poor patient outcome. Recently it has been found that therapeutic targeting of MYC using small molecule inhibitors can disrupt lipid metabolism through mitochondrial dysfunction [50, 51]. Although we have only assessed total lipid by 1H-MRS in tumor tissue, it is highly likely that both MYC/N amplified neuroblastoma and medulloblastoma have altered lipid signatures, and that therapeutic targeting of lipid metabolism may offer a potential avenue for directed treatment in these high risk cases. There is a known association between elevated lipids and poor outcome in non-MYC/N driven brain tumors [52], hence ongoing investigation of underlying lipid metabolism and the relationship to other metabolic pathways in cancer is essential.

### Retinoblastoma and medulloblastoma are most metabolically similar, whilst neuroblastoma is more distinct

The two tumors that appear most metabolically similar are retinoblastoma and medulloblastoma. It is evident that both contain large concentrations of metabolites reflecting both CNS neural origin and similarities in metabolic function during development. Despite this, these two tumors can be distinguished, with retinoblastoma containing relatively higher levels of metabolites that seem to reflect the increased energy demands of normal retina. Moreover medulloblastoma is distinguished by elevated PC, glycine and scylloinositol, markers of increased cellularity, high proliferation and poor clinical prognosis in brain tumors. Neuroblastoma had a distinct metabolic profile that was markedly different from the other two groups, with less taurine, very little glutamine and elevated myoinositol and glutamate. Although these embryonal tumors all derive from primitive neuroectodermal tissue, neuroblastoma arises from the neural crest rather than the neural tube and occurs throughout the sympathetic nervous system. Therefore it may not represent as closely the metabolic features seen in retinoblastoma and medulloblastoma which originate in the CNS. The developmental influences on tumor metabolism are also likely to be different outside of the CNS. Moreover, recent debate suggests that neural crest derived tumors should be considered separate entities from neuroectodermal tumors of the CNS despite similar histological features and origins [53].

Although a direct comparison with the underlying histology of each tumor is outside the scope of this work, there is typically more heterogeneity in the underlying neuroblastoma pathology, including a higher stromal component [4] not typically seen in medulloblastoma or retinoblastoma. Although we have not previously found intra-tumor heterogeneity to influence metabolic profile in neuroblastoma tissue [12], there is more underlying variation in metabolic profile between samples in the neuroblastoma group which may reflect heterogeneity in underlying histology. This will be examined in future work in the neuroblastoma cohort.

## MATERIALS AND METHODS

### Clinical data

Frozen tumor tissue was collected over a period from 1996–2016 from biopsied or surgically resected medulloblastoma, neuroblastoma and retinoblastoma patients and stored at –80 degrees. Tissue was obtained from the Birmingham Children’s Hospital tumor bank and the UK Children’s Cancer and Leukaemia Group (CCLG) tumor bank (neuroblastoma and retinoblastoma only). Consent was obtained for tissue banking for ethically approved research from the local Research Ethics Committee and the CCLG Biological Studies Committee (neuroblastoma and retinoblastoma). All medulloblastoma and retinoblastoma patients were treated locally at Birmingham Children’s Hospital, some neuroblastoma tissue was obtained from other treatment centers via CCLG. Pathology reports, and limited clinical and survival data were available for all cases (Table 1). The cohort of tumors under investigation represented a wide range of molecular and clinical stages. Of the 50 retinoblastoma patients, four had bilateral disease and the remaining had unilateral disease, and all cases were sporadic rather than familial. This cohort has been described previously [14]. The 39 medulloblastoma cases were distributed across the histological variants namely, large cell anaplastic, desmoplastic nodular, and classic. One medulloblastoma was MYC amplified, whilst two were MYCN amplified. The 70 neuroblastoma samples included cases from low and high stage disease (1, 2, 3, 4 and 4S). Eleven of the 53 stage 3 & 4 neuroblastoma were MYCN amplified.

### High-resolution proton magnetic resonance spectroscopy (1H-MRS)

Briefly, frozen tissue was weighed, trimmed to fit in either a 50ul or 12ul zirconium rotor and 5ul of internal standard was added (TSP Cambridge Biosciences, Cambridge, UK), before the rotor was topped up with deuterated water (Sigma-Aldrich, Dorset, UK). There were no samples where the volume of liquid was inaccurate or spilled during preparation hence no effect of this on subsequent metabolite quantification in any samples. 1H-MRS was undertaken on a Bruker Avance 500MHz spectrometer using a 4mm 3 channel HR-MRS z-PFG band probe (Bruker, Coventry, UK) at the Henry Wellcome Building for Biomolecular NMR at the University of Birmingham. All data was acquired over a period from 2011-2016. Tumor tissue was kept cold over dry ice during preparation and the temperature was maintained at 4°C whilst acquiring data to minimize metabolite degradation. Samples were spun at 4800Hz with a pulse-acquire sequence with 2s of NOESY presaturation for water suppression and a repetition time of 4s. The total acquisition time was 17 or 34 minutes depending on sample size and signal to noise ratio. The mean sample weight and standard deviation for each tumor group was 20 ± 10.2 for medulloblastoma, 21 ± 7.8 for retinoblastoma, and 22 ± 14 for neuroblastoma.

### Data pre-processing and quality control

All processing of raw 1H-MRS spectra was undertaken in Mestrenova v9.0.1 (Mestrelab Research, Santiago, Spain). Spectra were Fourier-transformed, manually phased, automatically baseline corrected and chemical shift referencing performed relative to creatine at 3.03ppm. All spectra were visually inspected prior to undertaking quantification and only samples with high signal to noise and well defined metabolite peaks were used for further analysis. Whilst there is the potential for inaccuracies in quantification due to the hydrophobic interactions between internal standard TSP and membrane bound constituents in tissue, quality control procedures ensured that this was minimized. All TSP peaks were reviewed to assess the signal and confirmed to consist of a narrow well defined singlet with a consistent peak shape and linewidth at 0.0ppm. Due to the rarity of the tumor tissue under investigation and the need to maximize its availability for other molecular analyses, it was not possible to check the histology of every tissue sample in this cohort after 1H-MRS. However, previous analysis of post-MRS samples in a limited number of retinoblastoma [14], and other neural tissue (unpublished) provides a clear indication of the presence of tumor tissue versus normal tissue. Highly elevated phosphocholine, glycine, and lactate along with the presence of lipids and low NAA was used as a marker of spectra containing predominantly tumor. Samples with a large amount of normal neural tissue typically contain a much higher ratio of creatine to choline, a high ratio of GPC to PC and choline, very high NAA and lower glycine than tumor tissue. These definitions are consistent with prior studies reported in the literature [19, 20, 26, 54]. All of the tumor spectra included this study were assessed using these criteria and had features consistent with tumor.

### Quantification of metabolite concentrations

The region from 0-4.7ppm was analyzed for metabolite concentrations. Metabolites were manually assigned according to published literature and the Human Metabolome Database (HMDB) [55, 56]. Automatic peak detection using the global spectral deconvolution algorithm within Mestrenova was applied to the spectra to detect peaks. Metabolite assignments used for quantification are provided in Supplementary Table 1. Quantification of metabolite concentrations was undertaken relative to the internal standard, TSP. Using the qNMR function within Mestrenova, the fitted area of the assigned metabolite peak was compared to the fitted area of the TSP at known concentration, and adjusted for the proton number of the metabolite and TSP to obtain concentration values. Broad resonances originating from the main lipids peaks from 0-6ppm were assigned as described previously [14], Supplementary Table 2, and quantified as described above. Lipid data is presented as the total sum of lipid. Metabolite and total lipid concentrations were normalized to the total sum of all metabolites within the sample. Metabolite ratios between selected metabolites were obtained using the raw pre-normalized concentration values.

### Statistical analysis

Mean metabolite concentrations and ratios were statistically analyzed in SPSS (NY, USA) using non parametric Kruskall-Wallis tests with a Bonferroni adjusted significance value of *p* = 0.0017, to correct for multiple comparisons across metabolites. Where significance was achieved, pair wise post-hoc comparisons were undertaken with the Mann-Whitney *U* test. Multivariate analysis was undertaken using Metabaloanalyst (Version 3.0) and R [57]. Unsupervised PCA and dendrogram cluster analysis were carried out with metabolite concentrations as input. Euclidian distance measure and Wards clustering method were used for dendrogram analysis. Inter-metabolite correlations and heat maps were calculated with Pearson’s correlation measure.

Pathway analysis was also undertaken in Metabaloanalyst, using the pathway topography analysis module. This analysis uses the structure of the pathway to evaluate the importance of the metabolites within the pathway, and identifies the most relevant altered pathways between different groups [57, 58]. For this analysis, the homosapiens pathway library was used, with the global test method to evaluate differences between each tumor. Relative betweeness centrality was used to evaluate the importance of each metabolite within the pathways. Pathway impact values, scored out of 1, are presented with significant *p* values calculated using the false discovery rate (FDR) which is adjusted for multiple comparisons.

## CONCLUSIONS

Despite the histological similarities and neural metabolic features shared by all three tumors, this work shows that neuroectodermal derived embryonal tumor groups can be distinguished by tissue metabolic profile. It is apparent that some aspects of metabolism reflect the known characteristics of each individual tumor type, whilst others reflect developmental processes and the metabolic function of the normal tissue that these tumors derive from. Several metabolites and biological pathways including taurine, glutamate/glutamine, and myoinositol have been identified that are of prognostic and diagnostic interest and require further investigation in these tumors. We have also identified a relationship between elevated MYC/N and lipids. Although we have not endeavored to directly link clinical or pathological features to metabolites across the cohort in this study, ongoing parallel work in individual tumor groups shows promise for untangling the relationship between molecular features and metabolism [37], as well as the potential to identify non-invasive biomarkers using *in vivo* magnetic resonance spectroscopy. This is the first study to undertake comparative metabolic analysis of this rare group of pediatric tumors and it provides valuable insight into our understanding of tumor metabolites in childhood cancers. This work furthers our understanding of the biological pathways of childhood tumors derived from the neuroectoderm and may aid in identification of pathways to target for the development of novel therapeutics in this group of tumors.

## SUPPLEMENTARY MATERIALS TABLES



## Abbreviations

-1H-MRS: High resolution magnetic resonance spectroscopy
3HB: 3-hydroxybuturate
CNS: Central nervous system
FDR: False Discovery Rate
GABA: gamma-Aminobutyric acid
GPC: Glycerophosphocholine
HMDB: Human metabolome database
NAA: N-acetylaspartate
PC: Phosphocholine
PCA: Principal components analysis
