# Supplementary material for: Metabolic profiling of the three neural derived embryonal pediatric tumors retinoblastoma, neuroblastoma and medulloblastoma, identifies distinct metabolic profiles
Source: Oncotarget. 2018 Jan 11;9(13):11336–51. doi: 10.18632/oncotarget.24168 (PMC5834290; doi:10.18632/oncotarget.24168)
Supplement: Supplementary file 1 [file oncotarget-09-11336-s001.pdf]

Metabolic profiling of the three neural derived embryonal pediatric tumors retinoblastoma, neuroblastoma and medulloblastoma, identifies distinct metabolic profiles

SUPPLEMENTARY MATERIALS

Supplementary Table 1: Metabolite assignment, mean metabolite concentrations per tumor group, and significance values

Identified metabolite	PPM assignment for quantification	Normalised mean concentration value			Kruskall-Wallis p-value for inter-tumour metabolite comparisons	Significant pairwise post-hoc comparisons (Mann-Whitney *U* test, $p < 0.0017$)		
		RB	MB	NB		RB vs MB	RB vs NB	MB vs NB
3-Hydroxybuturate *(1)*	1.20 (d)	0.015	0.002	0.012	* $p < 0.0001$	↑	*ns*	↓
Acetate *(2)*	1.92 (s)	0.004	0.003	0.007	ns, $p < 0.002$	*ns*	*ns*	*ns*
Acetone *(3)*	2.22 (s)	0.001	0.000	0.002	ns, $p < 0.101$	*ns*	*ns*	*ns*
Alanine *(4)*	1.48 (d)	0.046	0.036	0.038	ns, $p < 0.002$	*ns*	*ns*	*ns*
Ascorbate *(5)*	4.52 (s)	0.031	0.032	0.023	ns, $p < 0.002$	*ns*	*ns*	*ns*
Aspartate *(6)*	2.82 (dd)	0.006	0.005	0.018	* $p < 0.0001$	*ns*	↓	↓
Beta-D-Glucose *(7)*	4.62 (d)	0.003	0.002	0.006	ns, $p < 0.686$	*ns*	*ns*	*ns*
Choline *(8)*	3.20 (s)	0.007	0.014	0.016	* $p < 0.0001$	↓	↓	*ns*
Creatine *(9)*	3.03 (s)	0.049	0.068	0.031	* $p < 0.0001$	*ns*	↑	↑
GABA *(10)*	2.30 (t)	0.012	0.003	0.000	* $p < 0.0001$	↑	↑	*ns*
Glutamate *(11)*	2.34 (m)	0.058	0.055	0.130	* $p < 0.0001$	*ns*	↓	↓
Glutamine *(12)*	2.44 (m)	0.057	0.067	0.028	* $p < 0.0001$	*ns*	↑	↑
Glutathione *(13)*	2.55 (m)	0.020	0.019	0.019	ns, $p < 0.951$	*ns*	*ns*	*ns*
Glycerophosphocholine*(14)*	3.23 (s)	0.033	0.076	0.045	ns, $p < 0.260$	*ns*	*ns*	*ns*
Glycine *(15)*	3.55 (s)	0.009	0.006	0.014	* $p < 0.0001$	↓	*ns*	↑
Hypotaurine *(16)*	2.65 (t)	0.029	0.017	0.000	* $p < 0.0001$	*ns*	↑	↑
Isoleucine *(17)*	1.01 (d)	0.003	0.002	0.003	ns, $p < 0.003$	*ns*	*ns*	*ns*
Lactate *(18)*	1.31 (d)	0.364	0.230	0.262	* $p < 0.0001$	↑	↑	*ns*
Leucine *(19)*	0.95 (t)	0.013	0.006	0.015	ns, $p < 0.01$	*ns*	*ns*	*ns*
Myoinositol *(20)*	3.52 (dd)	0.010	0.086	0.149	* $p < 0.0001$	↓	↓	↓
NAA *(21)*	2.03 (s)	0.004	0.011	0.018	* $p < 0.0001$	↓	↓	*ns*
Phosphocholine *(22)*	3.22 (s)	0.029	0.098	0.049	* $p < 0.0001$	↓	↓	↑
Phosphoethylamine *(23)*	3.21 (s)	0.014	0.015	0.029	ns, $p < 0.247$	*ns*	*ns*	*ns*
Scylloinositol *(24)*	3.34 (s)	0.000	0.003	0.004	* $p < 0.0001$	↓	↓	*ns*
Serine *(25)*	3.84 (dd)	0.004	0.005	0.005	ns, $p < 0.162$	*ns*	*ns*	*ns*
Succinate *(26)*	2.40 (s)	0.004	0.002	0.003	* $p < 0.0001$	↑	↑	*ns*
Taurine *(27)*	3.42 (t)	0.165	0.128	0.064	* $p < 0.0001$	↑	↑	↑
Valine *(28)*	1.07 (d)	0.010	0.007	0.010	ns, $p < 0.002$	*ns*	*ns*	*ns*
Total Lipid	*Additional file 2*	1.240	0.717	2.380	* $p < 0.002$	*ns*	↓	↓

*Bonferonni corrected significance value of $p < 0.0017$ RB = retinoblastoma, MB = medulloblastoma, NB = neuroblastoma ns = not significantly different, Arrows indicate if the concentration is increased or decreased in the first tumor group relative to the second tumor group. Peak shape is described for each metabolite assigned at its respective PPM resonance: (s) metabolite is a singlet, (d) metabolite is a doublet, (dd) metabolite is a doublet of doublets, (t) metabolite is a triplet, (m) metabolite is a multiplet.

Supplementary Table 2: Lipid assignment

Identified lipid group	Proton contribution	PPM assignment for quantification
Lip 1	C**H**$_3$	0.90 ppm
Lip 2	C**H**$_2$)n	1.29a,b ppm
Lip 3	C**H**$_2$-CH$_2$=O	1.59 ppm
Lip 4	C**H**$_2$-CH=	2.03 ppm
Lip 5	CH$_2$-C**H**$_2$-C=O	2.26 ppm
Lip 6	=CH-C**H**$_2$-CH=	2.80 ppm
Lip 7	-C**H**=C**H**-	5.30 ppm
